# Noneczematous Contact Dermatitis

**DOI:** 10.1155/2013/361746

**Published:** 2013-09-15

**Authors:** Domenico Bonamonte, Caterina Foti, Michelangelo Vestita, Gianni Angelini

**Affiliations:** Department of Biomedical Science and Human Oncology, Section of Dermatology, University of Bari, Piazza Giulio Cesare 1, 70124 Bari, Italy

## Abstract

Irritant or allergic contact dermatitis usually presents as an eczematous process, clinically characterized by erythematoedematovesicous lesions with intense itching in the acute phase. Such manifestations become erythematous-scaly as the condition progresses to the subacute phase and papular-hyperkeratotic in the chronic phase. Not infrequently, however, contact dermatitis presents with noneczematous features. The reasons underlying this clinical polymorphism lie in the different noxae and contact modalities, as well as in the individual susceptibility and the various targeted cutaneous structures. The most represented forms of non-eczematous contact dermatitis include the erythema multiforme-like, the purpuric, the lichenoid, and the pigmented kinds. These clinical entities must obviously be discerned from the corresponding “pure” dermatitis, which are not associated with contact with exogenous agents.

## 1. Introduction

Allergic contact dermatitis (ACD) is a common cutaneous eczematous disorder caused by contact (either direct or aeromediated) with a range of environmental substances. Pathogenetically, ACD results from an immune reaction involving both innate and adaptive immunologic mechanisms. In particular, hyperreactive response to small chemicals (haptens) penetrating the skin depends on a series of events, such as haptens capability to activate and mobilize cutaneous dendritic cells (cDC), generation of hapten-epitopes for T-cell recognition, and hapten-cDC complex ability to prime effector T cells with skin homing proprieties [1–3]. In sensitized individuals, skin or systemic challenge with the specific sensitizer determines rapid recruitment of effector T cells, along with natural killer lymphocytes, which mediate tissue damage through release of proinflammatory cytokines and through hapten-loaded keratinocytes killing.

Clinics of ACD are generally polymorphic. Besides the classic eczematous form, in fact, different noneczematous clinical variants are possible [1, 4–6]. The causes for such variability in ACD clinical aspects are many (Table 1). According to our data (unpublished), considering >30.000 patch tested individuals for contact dermatitis, noneczematous forms are slightly more common (52%) than the classic eczematous one (48%). Various clinical patterns of noneczematous ACD have been described: some are linked to topical use of specific haptens and others more often dependent on allergens systemic administration (Table 2). The most represented forms are described as follows.

## 2. Erythema Multiforme-Like Contact Dermatitis

Of all noneczematous clinical variants, the erythema multiforme-like (or “contact erythema multiforme”) is the most common. It can be elicited by different substances, particularly exotic woods, medicaments, and ethylenediamine (Table 3).

### 2.1. Causes

*Woods and plants.* Among exotic woods, Brazilian rosewood (*Dalbergia nigra*), pao ferro (*Machaerium scleroxylon*), and *Eucalyptus saligna* are relevant as occupational causes of erythema multiforme-like eruption in carpenters, foresters, and cabinet makers. Antigens in pao ferro and Brazilian rosewood are crossreacting quinones, respectively, R-3, 4-di-methoxy-dalbergione, and R-4-methoxy-dalbergione [7, 8]. Literature also lists extraoccupational cases from wooden bracelet [9] and pendants [10] made of *D. nigra*. *M. scleroxylon* has been described to cause a similar eruption in hobbyists who handled this type of wood to build boxes [11].

Other reported causes of erythema multiforme-like reactions include *Artemisia vulgaris* [12, 13], poison ivy [14, 15], *Hypericum erectum* [16], and terpenes [17]. Tincture of capsicum caused an analogous reaction in a woman who used the concoction to treat her knee arthritis [18]. *Inula helenium*, contained in a mixture to treat back pain, has also induced erythema multiforme-like eruption, with positive patch tests to sesquiterpene lactone mix and alantolactone [19]. Notably, *Primula obconica* can also induce comparable eruptions [20–23]. We observed an erythema multiforme-like reaction in a plant nursery worker, who had handled plants of *P. obconica*. The dermatitis involved hands, forearms, and face. Patch tests were positive to primin (0.01% in pet), leaves and flower. Histology showed foci of hyperkeratotic orthokeratosis, mild spongiosis, exocytosis, and few isolated necrotic keratinocytes; at the superficial and mid dermis, a largely perivascular lymphocytic infiltrate was present [24].

*Topical Medicaments*. Numerous topical drugs are reported as cause of erythema multiforme-like contact dermatitis, the vast majority being antimicrobials. According to our observation, pyrrolnitrin can trigger this kind of eruption [25, 26]. Other causative drugs include sulfonamide [27, 28], promethazine [25], neomycin [25], mafenide acetate [29], ethylenediamine [25, 30], and mephenesin [31, 32]. Among nonsteroid anti-inflammatory drugs, phenylbutazone [33], bufexamac [34], and mofebutazone [35] have been reported. Among corticosteroids, budesonide [36] and triamcinolone acetonide [37] caused analogous reactions.

*Miscellany. *Erythema multiforme-like eruptions can be the expression of contact allergy to nickel [38–41] and cobalt [39]. 9-Bromofluorene induced a skin acute reaction in several chemistry students, who were exposed to the product during its synthesis [42, 43]. Finally, many other compounds have been associated to erythema multiforme-like reactions, although exceptionally [5, 6].

### 2.2. Clinical Features

Early lesions are eczematous in morphology and localized at the allergen contact site. After a 1 to 15 days delay, the erythema multiforme-like eruption follows, involving the area around the original lesions or rather extending to the whole cutaneous surface. The latter occurrence generally ensues systemic exposition to drugs which the patient had previously been sensitized to topically. Target-like, erythematovesicular, or urticarial lesions are characteristic. Resolution is slow-paced; these manifestations persist usually much longer than the original eczematous lesions (or sometimes appearing after regression of the latter). Itching sensation is also typically present in polymorphic reactions [1]. Patch tests generally elicit eczematous positive reactions, with the exceptional vesico-bullous or urticarial lesions.

Differential diagnosis is set out with true erythema multiforme (Table 4), the latter showing almost all target-like lesions with typical acral distribution and crops-like onset.

### 2.3. Histopathology

The histology is generally aspecific. Epidermis shows spongiosis and exocytosis. Mild upper dermis edema and perivascular lymphohistiocytic infiltration are noticeable. Vacuolar degeneration of basal cells is rarely present, while epidermal necrosis is very mild or absent. When bullae are present, they are intraepidermal [1].

The histopathology of true erythema multiforme shows frank epidermal necrosis and vacuolar basal cells degeneration, while bullae are subepidermal [1].

## 3. Purpuric Contact Dermatitis

This particular form of noneczematous contact dermatitis is of unusual observation, and many cases remain undiagnosed. The eruption evolves in several weeks after the withdrawal of the offending agent and resolves with more or less persistent pigmentation. The purpuric aspects of contact dermatitis, and the respective patch test reactions can be secondary to irritant, or more frequently allergic, mechanisms [44].

### 3.1. Causes

The most frequent causative factors are listed in Table 5. Certain components of rubber and textile are recurrently reported in the literature.

*Rubber. *First reported cases date back to 1968: 9 women developed purpura from cloth elastic inserts; in every instance patch tests turned positive to N-isopropyl-N-phenyl-paraphenylenediamine (IPPD), a rubber antioxidant [45]. Other 2 cases, showing diffuse purpuric reactions with negative bloodwork, were associated to IPPD and specifically to the use of rubber boots [46]. Fisher reported 3 cases, respectively, from rubber diving suit, elasticized shorts, and rubberized support leg bandage; in all 3 patients patch tests turned positive to IPPD [47, 48]. The author therefore fashioned the “PPPP syndrome,” defined as an ACD characterized by pruritus, petechiae, and purpura, caused by IPPD. IPPD also prompted similar eruption in a woman in the pattern of her brassiere [49] and in a man at rubber boots contact sites [50]. PPPP syndrome has also been described following use of orthopedic elastic bandages [51] and rubber gloves [52]; in the latter case patch tests were positive not just to IPPD but to N-cyclohexyl-N′-phenyl-paraphenylenediamine and N,N′-diphenyl-para-phenylenediamine as well.

*Textile.* From 1969 to 1972, Osmundsen gathered 167 cases of purpuric reactions from an optical whitener contained in washing powders [53, 54]. The petechial and itchy dermatitis interested those areas which are typically subject to tighter contact with clothes (armpits, arms, upper limbs folds, neck and thighs). The offending agent was Tinopal CH 3566, a mixture of 2 noncrossreactive pyrazolines (monochlorobiphenyl-pyrazoline and dichloro diphenyl pyrazole). Tinopal CH 3566 was used to bleach nylon fibers and caused a similar epidemic outbreak in Spain, where 103 were collected [55]. From that time on, the product was discontinued with no more cases reported. As of today, risk-free stilbene-based optical whiteners are employed.

A sailor developed generalized purpuric lesion with pigmentary outcomes at sites of contact with the military blue uniform. Patch tests evidenced positive reaction to Disperse Blue 85, while histology demonstrated Schamberg disease sign [56]. We directly observed a case of purpuric ACD to Disperse Yellow 27 (Serisol Fast Yellow 6DW), an azoic dye used in acetate and polyester fibers, a result of para-aminoacetanilide and paraphenylphenol. The dye was part of a pair of trousers inner lining, and the dermatitis, while interesting the whole skin surface, started from and was particularly manifest at the thighs. Thin layer chromatography from textile extract demonstrated only one component, Disperse Yellow 27. Histology proved traditional aspects of ACD, with lymphocytic infiltrate and intense perivascular edema, associated to noticeable erythrocyte extravasation [57]. Purpuric eruptions have also been described in a black hats vendor from paraphenylenediamine [58], in British soldiers from formaldehyde resins contained in kaki wool shirts [59], and in a man assigned to mixed wool-synthetic residues harvesting [60].

*Plants. Frullania *induced a diffuse purpuric reaction; histology showed signs of leukocytoclastic vasculitis; however, circulating immune complex and complement deposition assays were also positive [61]. *Agave americana *L, of Agavaceae family, can determine purpuric contact dermatitis with histological features of leukocytoclastic vasculitis [62]. We also observed a similar case, secondary to plant latex contact [63]. *Zea mais* (corn) has been shown to induce irritant purpuric phytodermatitis some hours after contact to green leaves. Patch, photopatch, and scratch tests with alcoholic extracts of different plant parts (leaves, trunk, efflorescences) all resulted negative [64]. Two-hour experimental exposition to 98% d-limonene resulted in a severe and several week persistent purpuric reaction 6 hours after contact [65]. 

*Miscellany.* Fiberglass can induce direct or aeromediated contact dermatitis, with pruriginous, 0,1–0,5 mm diameter, mostly follicular purpuric papules. Exposed and nonexposed areas are both affected, since these fibers are able to pass through clothing [66, 67]. Clothes contaminated by being washed together with fiberglass curtains can also induce purpuric dermatitis [68].

Vasculitic purpuric eruptions to Peru balsam [25, 69], ethylenediamine [70, 71], benzoyl peroxide [72], and proflavine [73] have also been reported.

### 3.2. Patch Tests Purpuric Reactions

 As is well known among those who practice dermatoallergology, petechial reactions to cobalt patch test, without edema, vesicles, and infiltration, can be observed. These are toxic in nature rather than allergic. Schmidt et al., in a 4-year time span, observed 123 cases (4.7%) of cobalt petechial reactions out of a total of 2594 patch-tested patients. Twenty-three patients were retested and developed new petechial responses in 60% of cases. Based on these authors data, the incidence of positive allergic reactions to cobalt was lower (2.9%) than the incidence of primary irritant reactions [74]. Judging on our practice, cases of petechial nonallergic reactions to cobalt and chrome are indeed rather numerous and frequently reproducible. 

### 3.3. Clinical Features

Purpuric contact dermatitis can be either toxic or allergic in nature. From a clinical-morphological perspective, differential diagnosis is not straightforward: both present palpable purpuric elements, evolve slowly and are followed by variably intense and persistent pigmentation. At times, clinical extension represents a useful feature in differentiating the 2 forms, the irritant being strictly limited to contact sites. Moreover, lesional elements resolve more rapidly and are less infiltrated in the irritant form compared to the allergic one.

Diffuse contact irritation from fiberglass must be discerned from scabies, eczema (prurigo-like), animal and vegetal acariasis, and if persistent, from Hodgkin disease. The anamnestic data of epidemic bursts in industries or bureaus (fibers dispersed from defective air conditioners) greatly aid diagnosis [75].

The allergic form of purpuric contact dermatitis generally features diffuse and polymorphic manifestations: papulopurpuric and papulovesicular lesions parallel classic eczematous foci. The latter are limited to the original contact site with the offending noxa. Secondary distant lesions can also present polymorphic or vasculitic aspects, as we have directly observed. Purpuric patch tests reactions are obviously vesicular and infiltrated [44].

### 3.4. Pathogenesis and Histopathology

The pathogenetic mechanism of purpuric contact dermatitis is currently unknown. Hemostasis or complement system alterations are not generally described in reported cases nor are immune complexes commonly isolated. In every case we observed, among which 3 severe cases from Peru balsam with frankly vasculitic and bullous lesions and various cases from ethylenediamine (in which the rash had followed systemic administration of aminophylline), specific laboratory exams fell into normal range [25, 44].

Since endothelial cells degeneration is evident at electron microscopy, a selective effect on these cells has been hypothesized. In detail, specific toxic or allergic substances as well as certain mechanical stimuli (fiberglass) would exhibit an affinity for vessels endothelium [47, 57, 58]. Alternatively, a primary lymphocytic reaction in response to the antigen at the perivascular site would free toxic lymphokines, ultimately responsible of endothelial damage [72].

Histopathology has been described, with comparable results, in most reported cases. In the epidermis, spongiosis and lymphocytic exocytosis are constant features, along with possible bulla formation. In the upper dermis the signs of leukocytoclastic vasculitis (vessels fibrinoid degeneration, edematous endothelium, scarce perivascular lymphomonocytic and neutrophilic infiltrate, erythrocytes extravasation, and karyorrhexis) are visible. The same features are present when examining a patch test response lesion (Table 6) [47, 74].

Bloodwork, histologic and patch test examinations are valid to differentiate the condition from vascular, hemostatic, and idiopathic purpuric affections.

## 4. Lichenoid Contact Dermatitis

A particularly uncommon form of noneczematous contact dermatitis presents with clinical features resembling those of lichen planus. It affects both skin and mucosal membranes.

### 4.1. Causes

Color developers, substances derived from paraphenylenediamine, are the most common cause of allergic contact lichenoid eruption. Among these compounds, Kodak CD2 (4-N, N-diethyl-2 methylphenylenediamine), Kodak CD3 (4-N-ethyl-N-2-methanesulfonylaminoethyl-2-methyl-phenylenediamine sesquisulfate monohydrate), Kodak CD4 (2-amino-5-N-ethyl-N-(hydroxyethyl)-aminotoluene sulfate), Ilford MI 210 (N-ethyl-N (5-hydroxy-amyl) para-phenylenediamine hydrogen sulphate), and Agfa TSS (4-amino-N-diethylaniline sulfate) [75].

Other cases of lichenoid contact dermatitis have been reported by Mandel, in 9 out of 11 workers with contact allergy to a color developer [76], and by Fry in 7 out of 20 patients with analogous sensitization [77]. High speed, black-and-white film processing implies the use of similar chemicals, which can induce lichenoid reactions [78]. As a general rule, the eruption from color developers spares the oral mucosa [79]. Cases from paraphenylenediamine in hair dyes [80], *P. obconica* [81], nickel [82], epoxy resins [83], aminoglycoside antibiotics [84], and methacrylic acid esters for industrial use [85] have also been described. Oral mucosae involving forms are due to copper [86], zinc [87], and mercury [88] contained in dental restorations.

### 4.2. Clinical Features

Eczematous lesions evolve or associate with papulous lesions with peculiar lilac-red gradation. The eruption mostly involves contact sites, later widely spreading with mucosal sparing. Course is prolonged and leaves variably intense pigmentary changes lasting up to some months. Lichenoid contact dermatitis has to be differentiated from lichen planus, with its characteristic papulous polygonal lilac lesions. The onset of lichenoid contact dermatitis is almost invariably acute and the eruption spreads rapidly. Frankly eczematous lesions at the primitive site are noticeable in many cases.

Positive reactions to patch tests are eczematous in nature, but might turn lichenoid.

### 4.3. Pathogenesis and Histopathology

Pathogenesis of contact lichenoid dermatitis is unclear. Systemic absorption of offending agents can elicit skin lesions far from the original site of contact. In 5 cases we observed (3 from color film developers and 2 from paraphenylenediamine), histology displayed lack of hypergranulosis, foci of moderate spongiosis, and focal basal stratum vacuolization. A patchy mononuclear infiltrate was evident in the upper dermis [86]. Basal cell vacuolization is the cause of incontinentia pigmenti, which could explain skin lesions peculiar color, a blend of red from flogosis with blue from dermal melanin.  Table 7 compares the different histopathological characteristic of lichenoid contact dermatitis and lichen planus.

## 5. Lymphomatoid Contact Dermatitis

This uncommon dermatitis manifests with the clinical features of plaque parapsoriasis or an early stage mycosis fungoides [1, 70]. There are no specific causing haptens [89–95], the most frequently reported being paraphenylenediamine, para-tertyl-butyl phenol resin, gold, ethylenediamine, and nickel. Patch test reaction to these is eczematous in nature and can persist for several days. Lymphomatoid contact dermatitis and mycosis fungoides alike present with infiltrative patches; the former, however, demonstrates a bright erythematous color and undefined margins.

Histology is crucial in differentiating the above two conditions. Spongiosis is much clearer, and exocytosis is typically lymphocytic in contact dermatitis. Mycosis fungoides instead shows atypical lymphocytes in focal abscess-like aggregations (i.e., the pathognomonic Pautrier's microabscesses) and a band-like subepidermal infiltration of large lymphoid cells with cerebriform nuclei (Table 8).

## 6. Pigmented Contact Dermatitis

Described by Osmundsen in 1970, it is a melanic primitive hyperpigmentation, usually observed in dark phototypes and mostly occupational [96]. The author observed an intense and bizarre skin hyperpigmentation due to contact with an optical whitener (Tinopal CH 3566) used in washing powders and made by a combination of two pyrazolone derivatives, as of now discontinued.

Clinically, involved sites were those of textile contact dermatitis, with brown-blue to grayish hyperchromia. The same occurred at patch test application sites. Histology evidenced melanin deposits inside and out melanophages in the upper dermis.

Pigmented contact dermatitis can also be prompted by azoic dyes. An epidemic outburst from contact to naphthol AS has been reported in a textile business [97]. Hyperpigmentation was noticeable in dark skinned individuals, while fair skinned ones showed the signs of classical eczema. Sudan I, Vacanceine Red [98], and Brilliant Lake Red R [99] are other offending colorants which have been reported. Isolated occupational cases from insoluble cutting oils [100], paraphenylenediamine [101], and other substances are also described (Table 9) [102]. Riehl's melanosis is nowadays also considered a pigmented contact dermatitis, mostly from cosmetic sensitizing fragrances and chemicals [103].

## 7. Pustular Contact Dermatitis

Pustules are usually associated with irritant reactions. Nevertheless, allergic pustular reactions are known from nitrofurazone [104], black rubber [105], and minoxidil [106]. The latter has been described in a woman who developed a vesicopustular eruption on the forehead after applying 2% minoxidil solution. Histology showed perifollicular lymphocytes, histiocytes, and eosinophils. Patch test response was erythematovesicopustular. Patch test was strongly eczematous in another case of pustular allergic contact dermatitis from isoconazole nitrate [107].

The implication of such rare pustular reactions remains uncertain. Pustules are sterile and transient and can displace subcorneally, as observed in a case from trichloroethylene [108].

### 7.1. Pustular Patch Test Reactions

Pustular reactions to contactants are frequently observed in patch test reading. Hjorth stated that atopics are predisposed to such reactions [109]. Metal salts, particularly nickel, copper, arsenic, and mercury represent the most common causes of these reactions, which are irritant in nature [110, 111]. As a matter of fact, pustular responses to nickel patch test are widely observed when testing atopics on lesional skin, with follicular papules, erythema, or lichenification [112]. This further supports the irritant nature of the phenomenon.

In subjects affected by atopic dermatitis, we often observed such pustular follicular reactions when patch testing with nickel but also with potassium bichromate. Pustules are always sterile, dry promptly, and resolve rapidly. Erythema is mild and the reaction is not pruriginous. Histology, documented in various cases, has always evidenced intraepidermial aggregations of neutrophils, without signs of lymphomonocytic exocytosis or spongiosis. We have always considered these reactions we directly observed irritant in nature [113, 114].

## 8. Dyshidrosiform Contact Dermatitis

Certain authors include this condition among noneczematous allergic contact forms [6]. In our opinion, this dermatitis retains frankly clinicohistologic eczematous aspects, and a proper differential diagnosis would have to be made with the endogenous eczema pompholyx. As per our observations, dyshidrosiform allergic contact dermatitis can be primitive or secondary [70, 115, 116]. The latter is defined as a contact sensitivity which complicates a preexisting primitive palmoplantar pompholyx. The latter tends to a chronic recurrent course, thus constituting a predisposing factor to occupational and extraoccupational contact allergy [117, 118]. From studies we carried out on 354 subjects with pompholyx genuine lesions, observed during a 5-year period, incidence of relevant positive patch tests reactions was 29.6%. Topical medicaments (used to treat the original pompholyx) and other substances among which paraphenylenediamine (31.5% positive reactions), chrome (25%), cobalt (10.2%), mercaptobenzothiazole (9.3%), nickel (6.5%), and para-*tert*-butylphenol formaldehyde resin (2.7%) were the most often implicated haptens. Patch tests relevance was related to specific occupational activities, use of peculiar gloves rather than shoes [115]. More recently, a study we conducted on 45 individuals affected by palmoplantar pompholyx confirmed an ACD incidence of 31% [116].

Primitive dyshidrosiform ACD is instead an expression of systemic contact allergy, of common observation in nickel sensitized patients. Oral challenge test with nickel reproduces the dyshidrosiform eruption in these subjects [119–122], although this phenomenon has not been widely confirmed [123, 124].

Table 10 designates differential diagnosis between dyshidrosiform ACD and pompholyx. Intense erythema and constant hand dorsum involvement in the former represent useful discerning characteristics. Histologically, spongiosis and exocytosis are much more marked in ACD than in pompholyx.

## Figures and Tables

**Table 1:** Factors determining the peculiar polymorphic clinical features of allergic contact dermatitis.

Eruptive polymorphism	
Evolutive polymorphism	
Causative agent	
Patient sensitizing level	
Way of exposition (cutaneous, systemic)	
Means of exposition (cutaneous direct, cutaneous aeromediated)	
Tissue structures targeted by the causative agent	
Anatomophysiology of the cutaneous region involved	
Causative agent possible concomitant irritant action	
Environmental factors (UV, temperature, humidity)	
Itching intensity variability	
Preexisting dermatitis underlying the overlapping contact allergy	

**Table 2:** Different types of noneczematous contact eruptions.

Erythema multiforme-like contact dermatitis
Purpuric contact dermatitis
Lichenoid contact dermatitis
Lymphomatoid contact dermatitis
Pigmented contact dermatitis
Pustular contact dermatitis
Dyshidrosiform contact dermatitis

**Table 3:** Causative allergens in erythema multiforme-like eruption.

Plants and woods	Medicaments	Miscellaneous chemicals
*Dalbergia nigra* (Brazilian rosewood)	Ethylenediamine	Brominated compounds
*Toxicodendron radicans* (poison ivy)	Pyrrolnitrin	Phenylsulphone derivatives
*Primula obconica *	Sulfamide	Epoxy resin
*Machaerium scleroxylon* (pao ferro)	Econazole	Formaldehyde
*Artemisia vulgaris *	Promethazine	Disperse blue 124
*Eucalyptus saligna* (gum)	Balsam of Peru	Trichloroethylene
*Inula helenium *	Scopolamine	Dinitrochlorobenzene
Capsicum	Mafenide acetate	Diphenylcyclopropenone
Terpenes	Proflavine	
Pyrethrum	Neomycin	
	Mephenesin	
	Vitamin E	
	Budesonide	
	Bufexamac	
	Clioquinol (vioform)	
	Ketoprofen	
	Triamcinolone acetonide	
	Idoxuridine	
	Phenylbutazone	

**Table 4:** Differential diagnosis between true erythema multiforme (EM) and erythema multiforme-like contact dermatitis.

Criteria	EM	EM-like contact dermatitis
Etiology	Viruses, bacteria, systemic drugs	Various topical chemicals

Clinical features	Erythematoedematous lesions with cockade appearance, sometimes bullous, with acral localization (face, hands, forearms, thighs)	Polymorphic lesions located peripherally to the contact site with the sensitizing agent

Fever	Often present	Absent

Mucosal involvement	Frequent	Rare

Histology	Epidermis: basal cells necrosis, subepidermal vesicobullae Dermis: edema, capillary vasodilation, vasculitis signs	Epidermis: spongiosis Dermis: edema, lymphohistiocytic infiltrate

Pathogenesis	Immunocomplexes	Type IV hypersensitivity

Patch tests	Negative	Positive

Course	Self-limited in 3 weeks	Favorable after allergen withdraw

**Table 5:** Causative agents in purpuric contact dermatitis.

Rubber compounds	Textile compounds	Plants	Miscellany
N-isopropyl-N′-phenyl-paraphenylenediamine	Optical whiteners (Tinopal CH 3566)	*Agave americana L*	Paraphenylenediamine
Mercaptobenzothiazole	Azoic dyes	*Zea mais *	Fiberglass
	Rubber compounds	*Frullania*	Peru balsam
	Formaldehyde resins	d-Limonene	Epoxy resin
			Oxyquinoline
			Proflavine
			Cobalt
			Benzoyl peroxide

**Table 6:** Histopathological characteristics of purpuric contact dermatitis (PCD) and vasculitis (V).

Criteria	PCD	V
*Spongiosis *	++	Negative
*Subpapillary edema*	+	++
*Leukocytoclasis *	Negative	++
*Erythrocyte extravasation *	+	+++
*Neutrophilic perivascular infiltrate *	+	+++
*Vasal involvement *	+	+++
*C3-direct immunofluorescence *	Negative	++

**Table 7:** Histopathological characteristics of lichenoid contact dermatitis (LCD) and lichen planus (LP).

Criteria	LCD	LP
*Spongiosis *	++	Negative
*Hypergranulosis *	−/+	+++
*Basal cells vacuolar degeneration *	+	+++
*Incontinentia pigmenti *	+	+++
*Civatte bodies *	Negative	++
*Dermal papillae *	Lengthened	Broaden, dome shaped
*Lymphohistiocytic infiltrate *	Mild perivascular	Band-like

**Table 8:** Histopathological characteristics of lymphomatoid contact dermatitis (LCD) and mycosis fungoides (MF).

Criteria	LCD	MF
*Spongiosis *	+++	+
*Exocytosis *	−/+++ Inflammatory cells	+++ Atypical lymphoid cells (microabscesses)
*Lymphocytic infiltrate *	Perivascular	Band-type
*Lymphocytes with cerebriform nuclei *	−/+	+++

**Table 9:** Causative agents in pigmented contact dermatitis.

Optical whiteners	Tinopal CH 3566
Dyes	Naphthol AS Sudan I Brilliant Lake Red Vacanceine Red Solvent orange 8

Cosmetics	Pigments: Pigment orange 3 Pigment red 3 Pigment red 49 Pigment red 53 Pigment red 64 Azoic solvents: Solvent orange 2 Solvent orange 8

Fragrances	Jasmine Hydroxycitronellal Ylang-ylang Patchouli Cananga

Antiseptics	Carbanilide

Miscellany	Formaldehyde Nickel Rubber *Primula obconica* Musk ambrette

**Table 10:** Differential diagnosis between dyshidrosiform ACD and pompholyx.

Characteristics	Dyshidrosiform ACD	Pompholyx
*Palms/soles *	+++	+++
*Hands/feet dorsum *	+++	+
*Erythema *	+++	+
*Hemorrhagic vesicles *	+	−
*Bullae *	+±	+/+++
*ACD primary locus *	Present	Absent
*Spongiosis *	+++	+
*Exocytosis *	+++	+
*Vesicles *	Minute	Large from coalescing
